# Role of adhesion receptor trafficking in 3D cell migration^☆^

**DOI:** 10.1016/j.ceb.2013.05.008

**Published:** 2013-10

**Authors:** Guillaume Jacquemet, Martin J Humphries, Patrick T Caswell

**Affiliations:** Wellcome Trust Centre for Cell-Matrix Research, Faculty of Life Sciences, University of Manchester, M13 9PT, UK

## Abstract

This review discusses recent advances in our understanding of adhesion receptor trafficking *in vitro*, and extrapolates them as far as what is currently possible towards an understanding of migration in three dimensions *in vivo*. Our specific focus is the mechanisms for endocytosis and recycling of the two major classes of cell-matrix adhesion receptors, integrins and syndecans. We review the signalling networks that are employed to regulate trafficking and conversely the effects of trafficking on signalling itself. We then define the contribution that this element of the migration process makes to processes such as wound healing and tumour invasion.

## Highlights

• Adhesion receptor trafficking makes a major contribution to cell migration in 3D.
• Integrin and syndecan receptors synergise to control signals for migration.
• Specific integrin heterodimers perform different roles during migration.

**Current Opinion in Cell Biology** 2013, **25**:627–632

This review comes from a themed issue on **Cell adhesion and migration**

Edited by **Carole A Parent** and **Orion D Weiner**

For a complete overview see the Issue and the Editorial

Available online 21st June 2013

0955-0674/$ – see front matter, © 2013 The Authors. Published by Elsevier Ltd. All rights reserved.

**http://dx.doi.org/10.1016/j.ceb.2013.05.008**

## Introduction

The ability of cells to translocate *in vivo* is a fundamental requirement for embryonic development and tissue homeostasis: it also makes an essential contribution to the aetiology of, and host response to, virtually every disease condition. An understanding of the complex molecular mechanisms that enable cell migration would therefore generate insights into a diverse range of biological processes, as well as offer the prospect of modulating aberrant movement. It is understandable therefore that there has been intense interest in defining the modes of migration employed by cells *in vivo*. Using a diverse range of model systems, from cultured cells on two-dimensional surfaces to intravital examination of xenografts, apparently distinct phenotypic processes have been described, including lamellipodial migration in 2D, and mesenchymal, amoeboid and lobopodial migration in 3D. In other articles in this issue, these different modes of migration are described in detail.

Regardless of mode, each type of cellular translocation shares some but not all, of the following features: receptor recognition of extracellular matrix (ECM) topography, formation and turnover of clustered adhesion signalling complexes, adaptation and deployment of dynamic cytoskeletal polymers, membrane uptake and delivery, and polarisation and spatial control of signalling. Whilst each of these features can be examined in isolation, it is likely that they are closely coordinated. Thus, adhesion complex clustering may be determined by ECM topography and/or cytoskeletal architecture, and membrane dynamics may control the sites where signalling occurs. A current aim is therefore to combine information obtained from highly reductionist approaches into high order models of migration.

## Integrins

Integrins are a major family of adhesion receptors. In mammals, 18 α and 8 β integrin genes encode polypeptides that combine to form 24 α,β heterodimeric receptors [1,2]. Both subunits are non-covalently associated, type I transmembrane proteins with large extracellular and mostly short cytoplasmic domains. The combined extracellular domains engage a range of extracellular matrix and cell surface ligands, whilst the cytoplasmic domains engage the actin cytoskeleton via a series of linker proteins [1,2]. Integrins enable cells to sample the topology and mechanochemical properties of their pericellular environment and respond by changing their position and differentiated state [1,2].

The regulation of integrin affinity by ligand and cytoskeletal proteins has been extensively studied, but in recent years the endocytic trafficking of integrins has emerged as a complementary mechanism through which the availability of integrins at the plasma membrane is controlled. Integrins are internalised via many of the best-characterised endocytic routes, and this dictates the ability of the receptors to promote cell migration in two and three dimensions (reviewed in [3,4]). For example, endocytosis controls the turnover of focal adhesions and therefore cell migration in 2D [5–7], whilst direct interactions between αvβ6 integrin and HAX-1 control receptor endocytosis, and have been shown to regulate invasion in 3D ECM [8].

Following endocytosis, integrins, like other cargo receptors, are sorted in early endosomes for degradation or recycling back to the plasma membrane [3,9,10]. As the degradative turnover of integrins is relatively slow, endocytic recycling is considered to play a major role in regulating the spatiotemporal availability of integrins at the plasma membrane. In this context, several studies have demonstrated that the recycling of integrins contributes to adhesion complex formation and migration in 2D [3,11,12].

**Figure 1:**
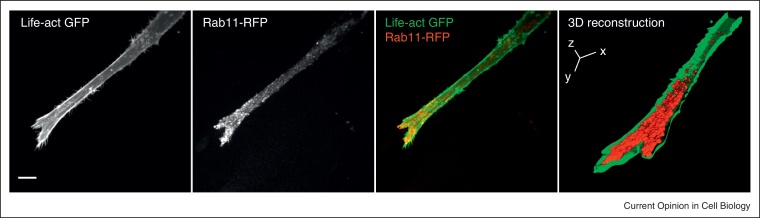
Rab11 positive vesicles accumulate at the front of cells migrating in 3D. Ovarian carcinoma cells (A2780) transiently expressing Life-act GFP and mCherry-Rab11 were plated on cell-derived matrices for 6 hours in the presence of cyclic RGD_fV_ peptide and imaged using a spinning disk microscope. Maximum projections of z-stacks are displayed (scale bar = 10 μm). The 3D reconstruction was performed using the Imaris software. To allow the visualisation of the Rab11 positive vesicles, only the bottom half of the actin stack was displayed.

Accumulating evidence suggests that trafficking integrins also play an important role in regulating invasive migration in 3D [13,14]. Indeed, in cells migrating in 3D-microenvironments, the vesicular regulators that control integrin trafficking accumulate towards the invasive front [15,16,17^•^] (Figure 1). It is notable that specific integrin heterodimers make different contributions to this process. For example, αvβ3 and α5β1 integrins bind to similar ligands, but can act antagonistically: whilst both integrins promote migration, they do so by eliciting different signalling responses and in fact mutually suppress each other [18]. Phosphorylation of rabaptin-5 by PKD promotes Rab4-dependent αvβ3 recycling, and this in turn promotes directionally persistent lamellipodial migration in 2D and invasion into 3D ECM in the absence of fibronectin (FN) [19,20^••^]. However, in the presence of FN, this αvβ3-recycling pathway suppresses invasive migration. This is because αvβ3, and αvβ3 recycling, inhibit the recycling and pro-invasive activity of α5β1 [16,19,20^••^]. When αvβ3 (or its recycling) is inhibited, or if cells express cancer-associated forms of mutant p53, α5β1 associates with the Rab11-effector Rab-coupling protein (RCP), and rapidly recycles to the plasma membrane to promote invasion into FN-rich ECM [16,20^••^,21]. Production of phosphatidic acid by DGKα promotes the recruitment of RCP to the front of invasive cancer cells via its C2 domain, resulting in localised trafficking in this subcellular region [17^•^]. Interestingly, RCP-driven α5β1 recycling does not influence the ability of the integrin to mediate attachment via its ligand FN: instead, α5β1 and RCP recruit receptor tyrosine kinases and regulate their trafficking and signalling to promote invasion [16,21,22].

α5β1 trafficking can also promote invasive migration in FN-rich ECM through Rab25, a Rab11 family member that is upregulated in aggressive ovarian cancer [23]. Rab25 binds directly to the cytoplasmic tail of β1 to direct α5β1 trafficking at the front of invading cells [15]. Here, endocytosed integrins are delivered to the Rab25 compartment at the cell front, and inactive integrins are trafficked directly back to the vicinal membrane [15]. Active α5β1 heterodimers are, however, trafficked via Rab25-positive late endosomes to lysosomes towards the rear of cell. Here, CLIC3, which is co-upregulated with Rab25 in a subset of aggressive ovarian and pancreatic cancers, promotes the recovery of α5β1 from lysosomes and recycling to the plasma membrane at the rear of the cell to facilitate invasion [24^••^]. Thus, Rab25 can coordinate process extension, by recycling unligated integrin to the cell front, with retraction by recycling active integrins to the cell rear where they can promote signals for forward movement.

## Syndecans

Syndecans are a small family of membrane-intercalated proteoglycans that serve as receptors for extracellular matrix ligands and growth factors [25]. In mammals, there are four members. It is remarkable that most ECM molecules possess both integrin-binding and syndecan-binding sites, and a clear synergistic relationship exists between these two families. For example, adhesion complex formation on several matrix ligands requires engagement of, and signalling via, a syndecan co-receptor. In this respect, syndecan-4 is the best-characterised family member, with its importance for migration *in vivo* being exemplified by the wound healing and angiogenesis defects observed in null mice [25]. In cells, cooperation between integrins and syndecan-4 has been demonstrated to regulate directional cell migration by dictating the spatiotemporal activation of the small GTPases Rac1, RhoA, RhoG and Arf6 [26–28,29^•^,30^••^].

Like integrins and growth factor receptors, syndecan function is regulated by endocytic trafficking [31^•^,32]. Whilst it has been suggested that syndecans can be internalised by macropinocytosis [33^•^], and that syndecan internalisation can be mediated via the binding of Rab5 to the syndecan-1 cytoplasmic domain [34], the mechanisms describing endocytosis of syndecans themselves are incompletely described. Syndecan recycling back to the plasma membrane has been shown to be dependent on a syndecan-syntenin-PIP2 association and the activity of the small GTPase Arf6 [32]. Interestingly, disruption of syndecan recycling by mutating the syntenin-PIP2 binding site triggers the accumulation of FGF receptor and β1 integrin to syndecan-containing endosomes. These observations suggest that syndecans could participate in the recycling of adhesion and growth factor receptors, possibly by trapping receptors into a specific endosomal compartment through their glycosaminoglycan chains [32].

Recent studies have indicated that syndecans are more than just passive cargos trafficked to and from the plasma membrane. Indeed, the syndecan-syntenin interaction has been shown to promote the formation of exosomes by recruiting ALIX [35^••^]. These data suggest that syndecans act as scaffolding platforms that recruit the machinery responsible for membrane budding and fission. Interestingly, exosome production was demonstrated to support tumour growth and metastasis, suggesting that syndecan functions could regulate these processes [36].

Syndecans have also been reported to regulate cell migration by controlling the internalisation and recycling of multiple receptors. Consistent with a regulatory role in receptor endocytosis, syndecans (in particular syndecan-4) have been shown to mediate the macropinocytosis of FGFR1 in response to FGF2 [33^•^], the clathrin-dependent internalisation of Wnt-receptor in response to R-spondin in Xenopus [37] and caveolar endocytosis of α5β1 integrin in response to H/0 (a soluble syndecan-4-binding fragment of FN) [29^•^]. In this context, the syndecan-4-mediated endocytosis of α5β1 has been shown to facilitate adhesion turnover and regulate directional cell migration in 3D microenvironments and efficient wound healing *in vivo* [29^•^].

**Figure 2:**
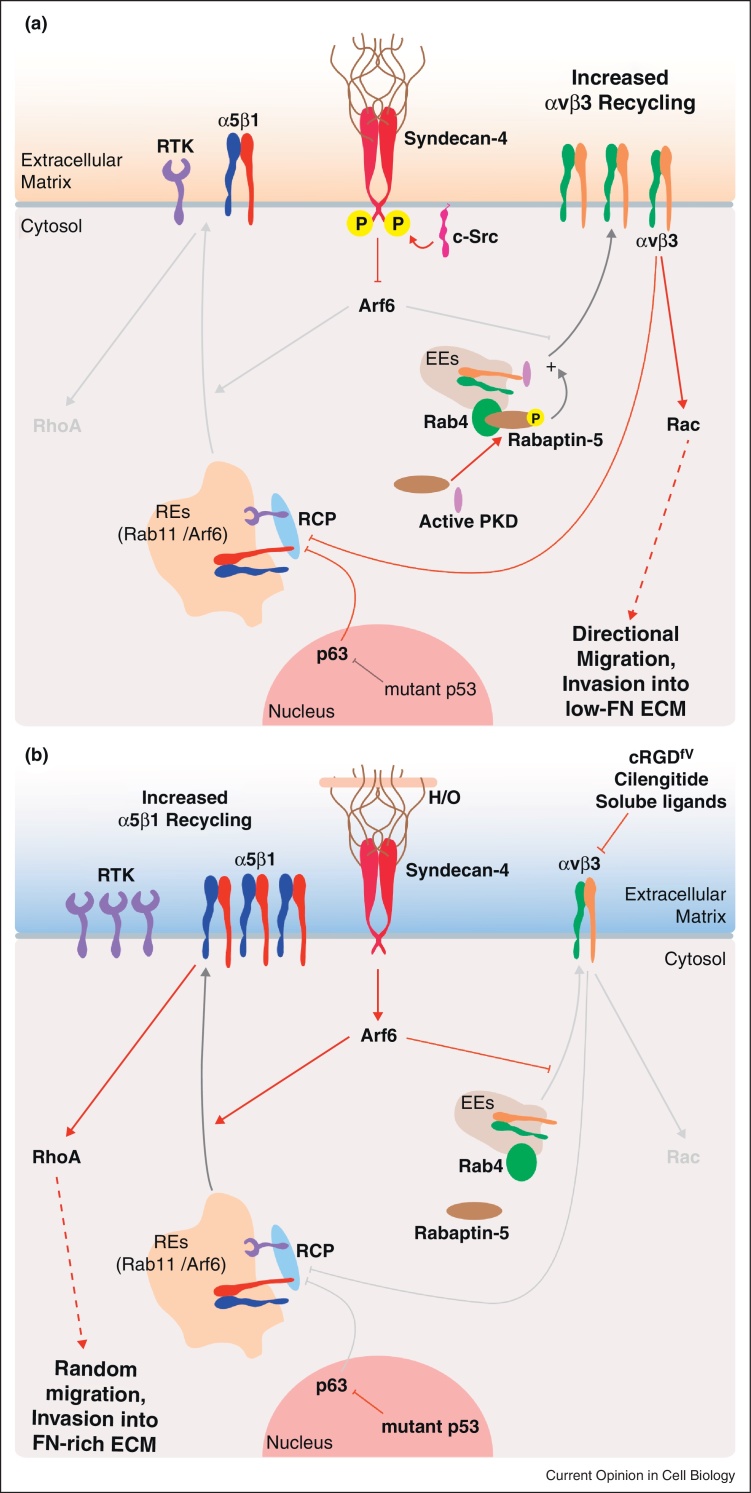
The mechanisms underlying the reciprocal nature of αvβ3 and α5β1 recycling. In many cell types, including cancer cells and fibroblasts, αvβ3 recycling suppresses the recycling of α5β1 to promote lamellipodial migration in 2D and invasion into ECM that lacks FN **(a)**. Intervening in the recycling of αvβ3, by manipulating αvβ3 directly, expressing mutant p53 (in cancer cells), or by influencing syndecan phosphorylation/engagement promotes the recycling of α5β1, and consequently a RhoA-ROCK dependent mode of random migration in 2D, and invasion into FN-rich ECM **(b)**. The studies summarised above are persuasive of the notion that the signalling and trafficking events governed by adhesion receptors such as integrins and syndecans should be viewed as a network, rather than individual, isolated events. Red arrows delineate signalling events whilst black arrows indicate endocytic trafficking.

In addition, syndecan-4 can dictate specificity of recycling between integrin heterodimers and therefore control the mode of cell migration [30^••^]. Src-dependent phosphorylation of syndecan-4 was shown to suppress Arf6 activity and to promote the recycling of αvβ3 integrin to the cell surface, leading to adhesion stabilisation and rapid directional migration on 2D substrates [30^••^]. Conversely, H/0-mediated stimulation of syndecan-4, or expression of a syndecan-4 construct that cannot be phosphorylated by Src, promoted Arf6 activation and the recycling of α5β1 integrin, resulting in adhesion turn-over and random migration on 2D substrates [30^••^]. Importantly, Src-mediated phosphorylation of syndecan-4 occurs in the conserved domain, present in all syndecan family members, and may represent a general mechanism whereby syndecans regulate receptor trafficking [30^••^]. Interestingly, Src can be activated by various receptors including integrins and growth factor receptors [38–40], allowing potential feedback loops within the recycling pathway. Furthermore, this recent study supports previous observations relating to the heterodimer specific signalling and trafficking to promote cell migration in 2D and in 3D [18,19,20^••^,41] (Figure 2).

Whether syndecans are involved in general receptor uptake or in the internalisation of specific receptors remains to be determined. As syndecans bind to an array of extracellular ligands, it will be important to assess whether specific syndecan ligands induce distinct internalisation pathways, or whether the internalisation route is dictated by the internalised receptor. A further priority will be to determine whether syndecans are internalised and trafficked together with, or separately from, these receptors.

From recent studies it is clear that syndecans, in particular syndecan-4, play a key role during cell migration by regulating the activation of various small GTPases and the trafficking of adhesion receptors. It remains to be elucidated whether these functions are independent or whether the syndecan-mediated temporal activation of small GTPases could be a consequence of the recycling pathway.

## Conclusion

Here, we have reviewed the recent advances that have altered perceptions of the role of adhesion receptor trafficking during cell movement. An emerging insight is the close connection between membrane dynamics and signalling, and we are beginning to clarify how these processes combine together to contribute to cell migration in a range of events *in vivo*. Whilst there is still much to be determined, some future perspectives can be discerned.

The approaches used to define the signalling events that are triggered by, and contribute to, receptor trafficking have in large part been defined by biochemical and immunocytochemical approaches. These techniques either lack precision or necessarily involve averaging of large cell populations. A priority for the future will therefore be improved precision, whether this involves localisation of signals to different membranes or pinpointing the sites at which vesicle budding and fusion occur.

A further priority will be to understand the variation in processes that underpin different modes of migration in different systems, and the mechanisms of switching that take place in relation to changes in cell phenotype and the mechanochemical environment of the cell. These studies will require a move to analysing ever more physiologically relevant, reconstituted 3D systems in which ECM composition, growth factors and mechanical properties have been reproduced, or the use of transparent organisms or intravital microscopy.

## References and recommended reading

Papers of particular interest, published within the period of review, have been highlighted as:

• of special interest
•• of outstanding interest
